# Inappropriate Hospital Admission According to Patient Intrinsic Risk Factors: an Epidemiological Approach

**DOI:** 10.1007/s11606-022-07998-0

**Published:** 2023-01-30

**Authors:** D. San Jose-Saras, J. Vicente-Guijarro, P. Sousa, P. Moreno-Nunez, M. Espejo-Mambié, J. M. Aranaz-Andres

**Affiliations:** 1 Universidad de Alcalá, Facultad de Medicina y Ciencias de la Salud, Departamento de Biología de Sistemas, Alcalá de Henares, Spain; 2 Servicio de Medicina Preventiva y Salud Pública, Hospital Universitario Ramón y Cajal, IRYCIS, Madrid, Spain; 3 Servicio de Medicina Preventiva y Salud Pública, Hospital Universitario Ramón y Cajal, IRYCIS, CIBER de Epidemiología y Salud Pública (CIBERESP), Madrid, Spain; 4 Facultad de Ciencias de la Salud, Universidad Internacional de La Rioja, Logroño, La Rioja Spain; 5 National School of Public Health, Public Health Research Center, Comprehensive Health ResearchCenter, CHRC, NOVA University Lisbon, Lisbon, Lisbon, Portugal

## Abstract

**Background:**

Inappropriate hospital admissions compromise the efficiency of the health care system. This work analyzes, for the first time, the prevalence of inappropriate admission and its association with clinical and epidemiological patient characteristics.

**Objectives:**

To estimate the prevalence, associated risk factors, and economic impact of inappropriate hospital admissions.

**Design and Participants:**

This was a cross-sectional observational study of all hospitalized patients in a high complexity hospital of over 901 beds capacity in Spain. The prevalence of inappropriate admission and its causes, the association of inappropriateness with patients’ intrinsic risk factors (IRFs), and associated financial costs were analyzed with the *Appropriateness Evaluation Protocol* in a multivariate model.

**Main Measures and Key Results:**

A total of 593 patients were analyzed, and a prevalence of inappropriate admissions of 11.9% (95% CI: 9.5 to 14.9) was found. The highest number of IRFs for developing health care-related complications was associated with inappropriateness, which was more common among patients with 1 IRF (OR [95% CI]: 9.68 [3.6 to 26.2.] versus absence of IRFs) and among those with surgical admissions (OR [95% CI]: 1.89 [1.1 to 3.3] versus medical admissions). The prognosis of terminal disease reduced the risk (OR [95% CI]: 0.28 [0.1 to 0.9] versus a prognosis of full recovery based on baseline condition). Inappropriate admissions were responsible for 559 days of avoidable hospitalization, equivalent to €17,604.6 daily and €139,076.4 in total, mostly attributable to inappropriate emergency admissions (€96,805.3).

**Conclusions:**

The prevalence of inappropriate admissions is similar to the incidence found in previous studies and is a useful indicator in monitoring this kind of overuse. Patients with a moderate number of comorbidities were subject to a higher level of inappropriateness. Inappropriate admission had a substantial and avoidable financial impact.

**Supplementary Information:**

The online version contains supplementary material available at 10.1007/s11606-022-07998-0.

## BACKGROUND AND OBJECTIVES

Inappropriate health care is defined as health care whose potential negative effects exceed the expected health benefit^1,2^, including inappropriate use, underuse (insufficient health care, with direct consequences for the patient), and overuse (the provision of unnecessary health care, with potential adverse events on patients and additional costs for the system).^3,4^

Resource overuse is one of the most expensive problems facing hospital management, accounting up to 101.2 billion dollars every year in the USA.^5^ The main cause of cost overrun in hospitals is inappropriate patient admissions. A recent meta-analysis estimated an incidence of 12.3% of inappropriate hospital admissions^6^, being associated with a younger patient with fewer comorbidities and with a lack of social support.^7,8^ However, there is a lack of previous studies that analyze the economic cost of inappropriate hospital admissions. Hospital overuse has been a major concern in the health systems worldwide, due to its high frequency and high economical cost, compared to other ways of overuse.^9,10^ In this context, the *Appropriateness Evaluation Protocol* (AEP), developed by German and Restuccia in 1981^11^, is the most accepted tool for assessing appropriateness of admissions and classifying its main causes of inappropriateness.^12–15^

Traditionally, the AEP has been used in studies that focused on social and non-clinical variables of the patient.^16^ However, this work approaches an epidemiological perspective considering patients’ intrinsic risk factors (IRFs) as medical comorbidities associated with the development of complications and adverse events related to health care. In a situation where resources are limited, an appropriateness assessment of health care services provided to patients is crucial to drive strategies for improving and maintaining a sustainable health care system.^3,6^ In this context, this study had a triple purpose: (1) to provide a prevalence value for inappropriate admission; (2) to analyze how patients’ IRFs could influence the appropriateness of hospital admissions; and (3) to estimate the economic impact of this inappropriateness.

## DESIGN AND PARTICIPANTS

### Study Design and Sample Size

This was a cross-sectional, descriptive, observational study that included the total of hospitalized patients in a high complexity hospital at a specific time (the second week of May 2019). The hospital was in Madrid (Spain), with a capacity of 901 beds and 45 operating rooms.

This study was conducted simultaneously and applying the same inclusion criteria as other cross-sectional patient safety studies, such as the Study on Patient Safety in Hospitals in the Community of Madrid (ESHMAD)^17,18^, based on the methodology proposed by Brenan et al. in *The Harvard Medical Practice Study*^19^, and the Prevalence of Nosocomial Infections in Spain (EPINE) study^20^, which has been carried out annually since 1990 in all high complexity hospitals in Spain and whose protocol was adapted by the ECDC to carry out the Point Prevalence Survey of Healthcare-associated Infections at the European level^21^, making it possible to calculate spatial-temporal trends in the prevalence of health care-associated infections. Based on this structure, a cross-section of patients was taken from each hospital ward on a date other than the first and last working days of the week to avoid overrepresentation of admissions or discharges. The following exclusion criteria were established: (1) patients admitted to the hospital or the emergency department on the same day as the start of the study (according to the criteria of the same patient safety studies); (2) patients hospitalized in the psychiatric, pediatric, or obstetric areas (due the inapplicability of the measurement instruments available).

### Measurement Instruments

The prevalence of appropriateness was measured using the *Appropriateness Evaluation Protocol* for admissions (AEP).^11^ This tool included more than 15 items referring to the clinical situation of the patient and the clinical care provided, such that, with the completion of one item, the admission was considered appropriate. One reviewer obtained the information to complete the protocol through clinical records. The adaptation used is included in the Supplementary Materials (Figure S1). For patients with an inappropriate admission, the cause was recorded as ‘Unnecessary admission’, ‘Patient needs institutional care of a lesser standard than an acute hospital’, ‘Premature admission’, or ‘Other’, using the definitions of the protocol.

### Variables

The primary study variable was admission inappropriateness, measured by the AEP. The cause of inappropriateness was recorded according to protocol.^11^

The following patient-related variables were considered: age; sex; IRFs; Charlson comorbidity index; reason for patient discharge (‘death’, ‘transfer’, or ‘discharge’); and prognosis of the disease leading to admission, classified as per the validated MRF2 form used in the ESHMAD study (‘full recovery to baseline’, ‘recovery with residual disability’, and ‘terminal illness’).^17,18^

The IRFs were the clinical data of patients associated with the development of complications and adverse events related to health care that were collected on admission. These were collected on admission and logged as dichotomous variables, considering the ‘presence’ or ‘absence’ of the factor, based on the criteria established by the ESHMAD^17^ and EPINE^20^ studies. The following variables were included: renal failure (clinical diagnosis or creatinine values higher than 1.7 mg/dl in previous or admission blood tests), sensory deficit prior to admission, previous mobility impairment, hypoalbuminemia (albumin lower than 3 g/dl in previous or admission blood tests), diabetes mellitus (clinical diagnosis or blood glucose higher than 145 mg/dl in blood tests on admission), obesity (BMI > 30), active smoking, cardiovascular disease (clinical diagnosis), neoplasia (in the previous five years), chronic lung disease (clinical diagnosis), cirrhosis (clinical diagnosis), coma (disturbance of consciousness on admission), neutropenia (neutrophil count <1000), immunodeficiency (diagnosis of primary or secondary immunodeficiency, neutrophil count <500 or HIV+ patients with CD4 count < 200), and preadmission pressure ulcers.

The health care variables included the admission department (medical, surgical, or intensive care unit (ICU)), admission type (urgent or scheduled), and the patient’s total length of hospital stay (days).

### Statistical Analysis

The association between inappropriateness and the main epidemiological variables was assessed using the *χ*^2^ hypothesis test or Fisher’s exact test for qualitative variables (depending on whether the variables were parametric or nonparametric) and the Student or Mann–Whitney *U* test for quantitative variables, after ruling out a normal distribution of the variable with the Shapiro–Wilk test. A statistical association was considered significant if *p*<0.050.

A multivariate predictive model was then developed using backward logistic regression of inappropriateness, eliminating the least significant variables and retaining those most associated (with *p*<0.100). Bootstrap techniques were used to correct for model overoptimism, and goodness-of-fit was analyzed using the Hosmer–Lemeshow test.

### Economic Analysis

For the cost analysis, the per day cost per stay per care unit was obtained using monetary equivalents provided by the center’s accounting department based on the costs for 2019. The cost overrun was estimated based on avoidable days of stay resulting from inappropriate admissions. The transfers of each patient were individually analyzed and considered for the estimation of the total cost of the avoidable days of hospitalization. Five indicators were estimated: (1) total, mean, and median of days of avoidable hospitalization per episode; (2) total economic cost per episode, according to the total avoidable days and the cost per stay per care unit; (3) total economic cost adding up the cost of each episode; (4) mean and median of the economic cost per patient per day; (5) total economic cost per day dividing the total cost and the mean of avoidable hospitalization days. After estimating the total economic cost per day, we performed an extrapolation of our data to the 70 hospitals of similar characteristics in Spain, and a total cost by year was calculated.

**Figure 1:**
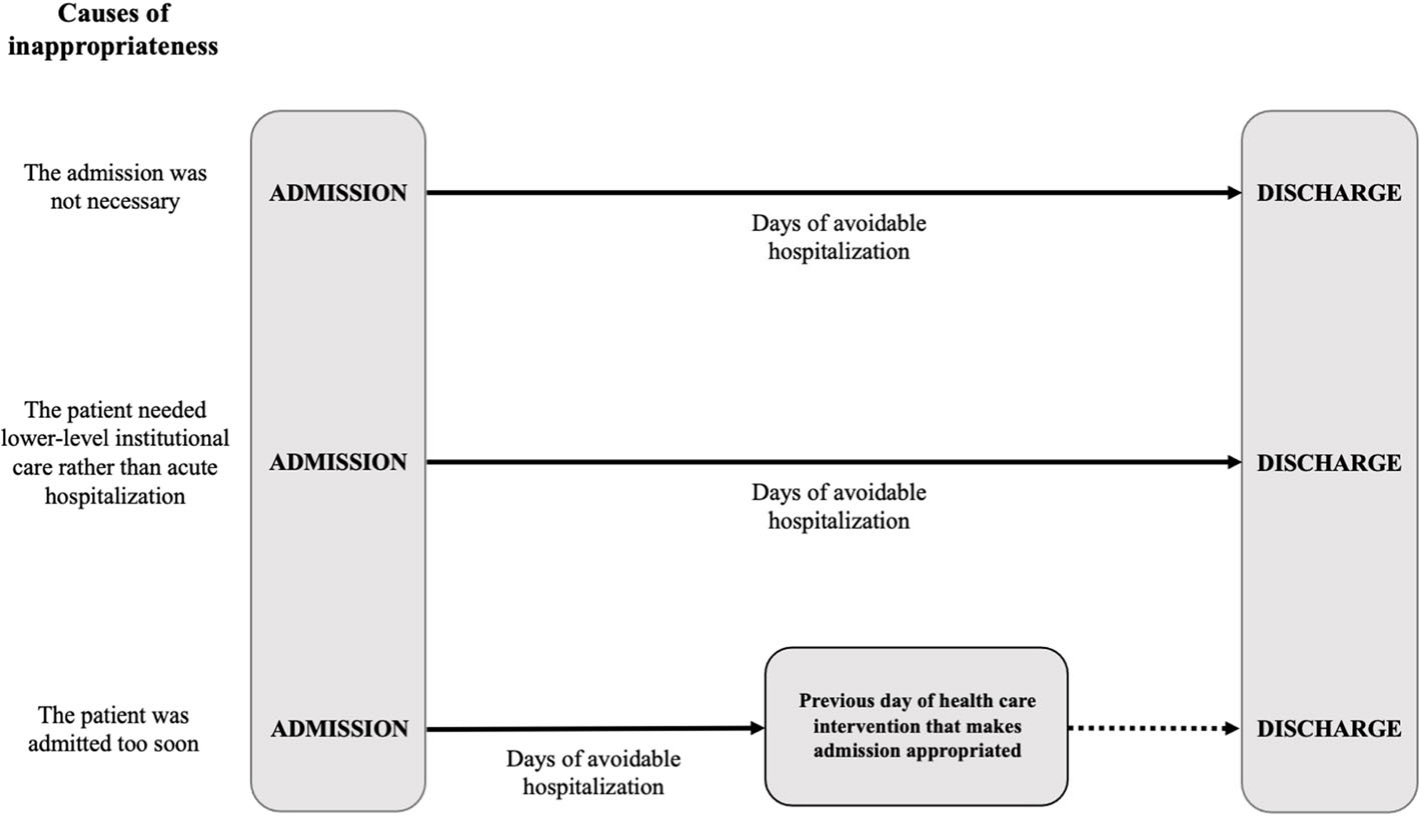
Diagram to estimate avoidable days of hospitalization per inappropriate admission by cause**.**

In the case of admissions that could have been managed as outpatients or did not require hospitalization in an acute unit, the entire hospital stay was considered avoidable, so the financial cost was calculated as the product of the mean cost of hospitalization per day in the care unit and the total number of days of hospitalization. For patients with premature admission, the days before the day prior to their intervention were classified as inappropriate. Admissions classified as inappropriate for reasons other than the above were analyzed individually. Financial cost was estimated in a stratified manner according to the cause of inappropriateness (Fig. 1).

The statistical analysis was carried out with STATA *Statistical* software, version 16 (StataCorp. 2019. College Station, TX: StataCorp LLC).^22^

### Ethics Committee

The ESHMAD study from which the patient sample was obtained was approved on 19 March 2019 by the Ethics Committee of the Hospital Universitario Ramón y Cajal (reference 057/19).

This inappropriate admissions study was approved on 3 March 2022 by the Research Ethics Committee of the International University of La Rioja (reference PI: 006/2022).

## RESULTS

### Characteristics of the Sample

**Figure 2:**
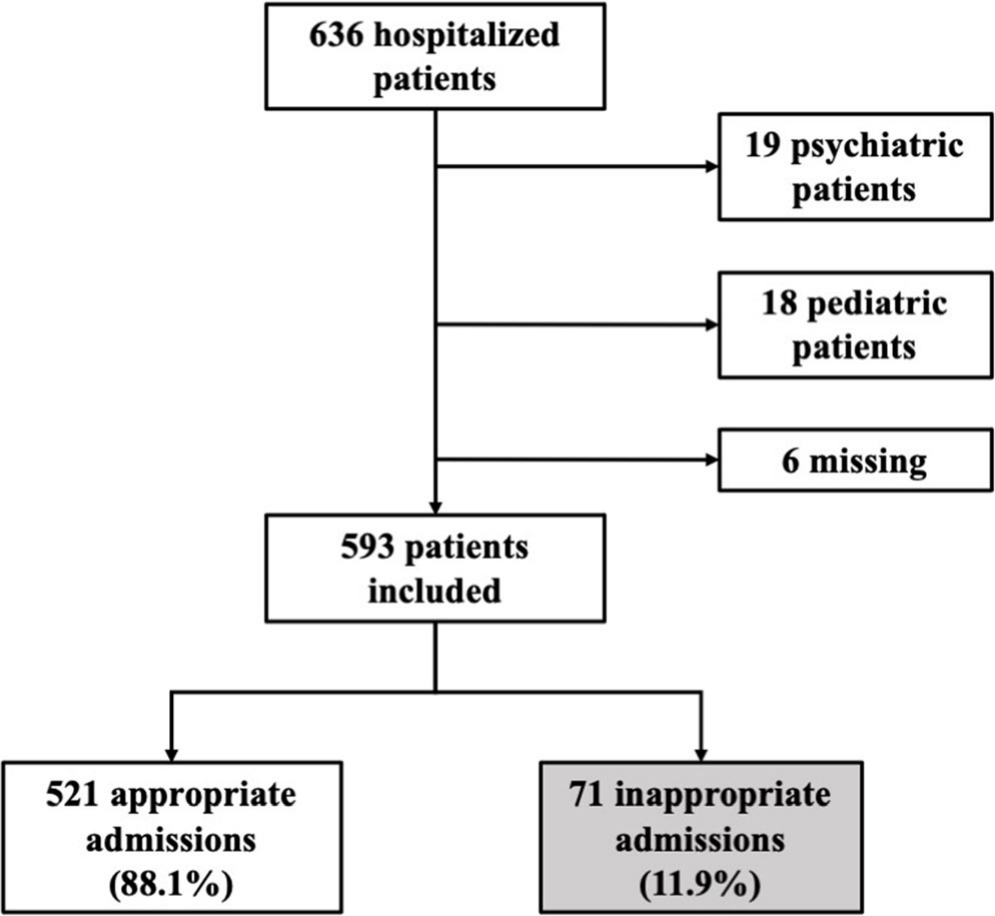
Flow diagram.

A total of 636 patients were hospitalized at the time of the study. Of these, 593 patients met the inclusion criteria. There were six losses due to transcription errors (Fig. 2). The median patient age was 72 years. Male patients accounted for 52.3%. A total of 54.6% of the sample had a prognosis of full recovery to baseline status for their main illness. The mean and median Charlson comorbidity index score were 3.0. Death occurred in 39 hospitalized cases.

A total of 66.1% of the patients were admitted from emergencies. The mean length of stay was 20.1 days, and the median was 11 days; 48.6% of patients were hospitalized for medical services (Table 1).

**Table 1:** Patient Characteristics

	Total sample	Prevalence of inappropriateness		*p*-value
	*n* (%)	*n*	% (95% CI)	
Age*				
Mean (SD)	69.1 (16.8)	69.2 (16.2)	65.4 to 73.0	0.995
Median (IR)	72 (58 to 82)	72.0 (59 to 81)	NA	
Sex				
Female	283 (47.7)	33	11.7 (8.2 to 16.0)	0.823
Male	310 (52.3)	38	12.3 (8.8 to 16.4)	
Number of intrinsic risk factors				
0	37 (6.2)	1	2.7 (0.0 to 14.1)	0.036**
1	83 (14.0)	14	16.9 (9.5 to 26.7)	
2	112 (18.8)	19	16.9 (10.5 to 25.2)	
≥ 3	361 (60.9)	37	10.2 (7.3 to 13.9)	
Prognosis of main illness				
Complete recovery to baseline status	323 (54.6)	39	12.1 (8.7 to 16.1)	0.003**
Recovery with residual invalidity	176 (29.7)	29	16.5 (11.3 to 22.8)	
Terminal illness	93 (15.6)	3	3.2 (0.1 to 9.1)	
Charlson comorbidity index score*				
Mean (SD)	3.0 (2.2)	2.6 (2.0)	2.1 to 3.0	0.075
Median (IR)	3.0 (1 to 4)	2.0 (1 to 4)	NA	
Death				
No	554 (93.4)	70	12.6 (10.0 to 15.7)	0.072
Yes	39 (6.6)	1	2.6 (0.0 to 13.5)	
Type of service				
Medical	288 (48.6)	22	7.6 (4.8 to 11.3)	<0.001***
Surgical	274 (46.2)	49	17.9 (13.5 to 22.9)	
Intensive care unit	31 (5.2)	0	NA	
Administrative status of admission				
Urgent	392 (66.1)	34	8.7 (6.1 to 11.9)	<0.001***
Scheduled	201 (33.9)	37	18.4 (13.3 to 24.5)	
Total length of stay (days)*				
Mean (SD)	20.1 (25.0)	16.4 (14.4)	12.6 to 20.1	0.838
Median (IR)	11 (6 to 24)	12 (6 to 19.5)	NA	
Total	593 (100)	71	11.9 (9.5 to 14.9)	

*SD*, standard deviation; *IR*, interquartile range; *NA*, not applicable

P for difference of percentages, chi^2^ test (for parametric test conditions) and Fisher’s exact test (for nonparametric test conditions)

P for quantitative variables tested by the Mann–Whitney *U* test because normality criteria were not used

*Age, Charlson comorbidity index score, and total length of stay were managed as numerical variables. For them, the first column contains the mean and median with SD and IR of the whole sample; the second column contains the mean and median with SD and IR of the group of patients with inappropriate admission; the third column contains the confidence intervals of the means in patients with inappropriate admission

**p<0.05; ***p<0.001

Approximately 60.9% of patients presented ≥ 3 IRFs. Among the IRFs, cardiovascular disease was present in 57.2% of the sample, followed by mobility issues (38.3%) and a history of neoplasia (34.1.6%). The distribution of IRFs is shown in Table S1 in the Supplementary Materials.

### Prevalence of Inappropriateness and Associated Epidemiological Characteristics

Seventy-one admissions were classified as inappropriate (11.9%, 95% CI: 9.5 to 14.9). The prevalence of inappropriateness was significantly higher among patients with a prognosis of recovery with residual disability (16.5% versus 12.1% among patients with a prognosis of full recovery; *p*=0.003), among those with scheduled admissions (18.4% versus 8.7% among those with urgent admissions; *p*<0.001), among those with surgical admissions (17.9% versus 7.6% among those with medical admissions; *p*<0.001), and among patients who had one or two IRFs (16.9%, respectively, versus 2.7% among those with 0 IRFs; *p*=0.036) (Table 1). No admission was classified as inappropriate in the intensive care unit. Individual analysis of each IRF can be consulted in Table S1 of the Supplementary Material.

### Causes of Inappropriateness and Multivariate Analysis

The main cause of inappropriate admission was premature patient admission (56.3%), followed by the potential to treat the patient’s diagnostic or therapeutic needs as an outpatient (33.8%).

Among the patients with scheduled admissions, the most frequent cause of inappropriate admission was premature admission (78.4%), followed by outpatient management (10.8%). Among patients with urgent admissions, the most frequent cause was the possibility of outpatient care (58.8%), followed by premature admission (32.4%). There were statistically significant differences in the causes of inappropriateness according to the type of admission (*p*<0.001) (Table 2).

**Table 2:** Causes of Inappropriate Admission

	Total	Scheduled		Urgent		*p*-value
	*n* (%)	*n*	% (95% CI)	*n*	% (95% CI)	
Premature admission	40 (56.3)	29	78.4 (61.8 to 90.2)	11	32.4 (17.4 to 50.5)	<0.001*
Admission was not necessary	24 (33.8)	4	10.8 (3.0 to 25.4)	20	58.8 (40.7 to 75.4)	
The patient needed lower-level institutional care rather than acute hospitalization	4 (5.6)	2	5.4 (0.6 to 18.2)	2	5.9 (0.7 to 19.7)	
Other	3 (4.2)	2	5.4 (0.6 to 18.2)	1	2.9 (0.0 to 15.3)	
**Total**	71 (100)	37	52.1 (39.9 to 64.1)	34	47.9 (35.9 to 60.1)	

P for difference of percentages: Fisher’s exact test (for nonparametric test conditions)

*p<0.001

A greater number of IRFs was associated with inappropriateness adjusted by age, prognosis of the main illness, type of service and administrative nature of the admission; the increase was greater among patients with one IRF (OR [95% CI]: 9.68 [3.6 to 26.2] versus the absence of IRFs), followed by patients with two IRFs (OR [95% CI]: 9.58 [2.1 to 42.8] versus the absence of IRFs) and patients with ≥ 3 IRFs (OR [95% CI]: 7.73 [2.0 to 29.6] versus the absence of IRFs). The inappropriateness was also associated with admission to the surgical area (OR [95% CI]: 1.89 [1.1 to 3.3] versus admission to the medical area). The prognosis of terminal disease reduced the risk of inappropriateness (OR [95% CI]: 0.28 [0.1 to 0.9] versus full recovery based on baseline condition).

The model calibration was assessed using the Hosmer–Lemeshow test, obtaining a *p*=0.304, with no difference between the observed and expected findings (Table 3).

**Table 3:** Multivariate Logistic Regression of the Inappropriateness of the Admission

	Total, *n* (%)	Inappropriateness by group, *n* (%)	Odds ratio	CI 95%	*p*-value
Age					
Increase of one year, mean in years (SD)	69.1 (16.8)	69.2 (16.2)	1.00	0.9 to 1.0	0.463
Sex					
Female	283 (47.7)	33 (11.7)	1.00	NA	NA
Male	310 (52.3)	38 (12.3)	1.19	0.7 to 2.1	0.541
Number of intrinsic risk factors					
None	37 (6.2)	1 (2.7)	1.00	NA	NA
1	83 (14.0)	14 (16.9)	9.68	3.6 to 26.2	<0.001**
2	112 (18.8)	19 (16.9)	9.58	2.1 to 42.8	0.003*
≥ 3	361 (60.9)	37 (10.2)	7.73	2.0 to 29.6	0.003*
Prognosis of main illness					
Complete recovery to baseline status	325 (54.6)	39 (12.0)	1.00	NA	NA
Recovery with residual invalidity	177 (29.7)	29 (16.4)	1.61	0.9 to 2.9	0.127
Terminal illness	93 (15.6)	3 (3.2)	0.28	0.1 to 0.9	0.034*
Admission area					
Medical	288 (48.6)	22 (7.6)	1.00	NA	NA
Surgical	274 (46.2)	49 (17.9)	1.89	1.1 to 3.3	0.027*
Intensive care unit	31 (5.2)	0	NA	NA	NA
Administrative status of admission					
Scheduled	392 (66.1)	34 (8.7)	1.00	NA	NA
Urgent	201 (33.9)	37 (18.4)	0.58	0.3 to 1.0	0.050
Charlson comorbidity index score					
Increase by a unit, mean (SD)	3.0 (2.2)	2.6 (2.0)	0.91	0.76 to 1.09	0.326
Constant	NA	NA	0.01	0.00 to 0.06	<0.001**

*95% CI*, 95% confidence interval; *p-value*, *p*-value estimated in the logistic regression; *NA*, not applicable

Multivariate analysis adjusted for age, number of intrinsic risk factors, prognosis of major disease, type of service and type of admission

*p<0.05; **p<0.001

### Financial Analysis

Inappropriate admissions accounted for a mean and median of 7.9 and 4 days, respectively, as well as a total of 559 avoidable hospital days. The mean cost per day per inappropriate admission to the hospital was €17,604.6/day, with a total additional cost overrun for avoidable hospital days of €139,076.4 (Table 4). Extrapolating this data to the 70 hospitals with more than 500 beds in Spain, inappropriate admission caused a daily cost overrun of €1.2 million and an annual economic loss of €449.8 million.

**Table 4:** Financial Cost of Inappropriate Admissions

	*n* (%)	Inappropriate days of hospitalization			Financial cost	
		Mean (SD)	Median (RI)	Total	Mean cost per patient*	Total
Cause of inappropriateness						
The admission was not necessary	24 (33.8)	15.2 (10.2)	14.0 (10 to 17)	365	€259.5/day	€94,715.3
The patient was admitted too soon	40 (56.3)	2.6 (3.5)	1.0 (0.6 to 3)	106	€237.0/day	€25,025.5
The patient needed lower-level institutional care rather than acute hospitalization	4 (5.5)	20.8 (8.5)	22.5 (14 to 27.5)	83	€215.8/day	€17,915.0
Other	3 (4.1)	1.7 (2.1)	1.0 (0 to 4)	5	€284.2/day	€1420.6
Clinical admission area						
Medical	22 (30.1)	14.2 (12.0)	11.5 (5.5 to 18)	312	€243.8/day	€76,066.7
Surgical	49 (67.1)	5.0 (6.6)	1.5 (1 to 8)	247	€255.6/day	€63,009.7
Administrative status of admission						
Urgent	34 (47.9)	11.7 (9.6)	11.0 (5 to 16)	397	€243.8/day	€96,805.3
Scheduled	37 (52.1)	4.4 (8.0)	1.0 (0.2 to 2)	162	€261.6/day	€42,271.1
Total	71 (100)	7.9 (9.5)	4.0 (1 to 13)	559	€249.0/day	€139,076.4€

*NA*, not applicable

*Average cost of inappropriate days of admission was calculated from length of stay in days added to the cost per day of admission in each care unit

Inappropriate admissions to the medical area accounted for the highest financial cost, followed by admissions to the surgical area (€76,066.7 and €63,009.7, respectively). Inappropriate emergency admissions accounted for double the financial cost compared to scheduled admissions (€96,805.3 versus €42,271.1).

## DISCUSSION

We analyzed 593 inpatients and found an inappropriate admission rate of 11.9%. Intrinsic risk factors (particularly 1 or 2 IRFs) and surgical admissions were associated with inappropriate admission. The most common type of inappropriate admission was premature admission (56.3%), followed by the possibility of managing the patient in an outpatient setting (39.3%). Inappropriate admission led to 559 avoidable days in hospital and a direct cost overrun of €139,076.4.

Measuring inappropriate admission is complex due to the multiple factors that influence it. There is no single tool for assessing appropriateness; in some cases, panels of experts are consulted.^23,24^ The AEP^11^ has been adapted and validated in many countries^25–27^, including Spain^28^, and its use is still spreading^29^, so the results obtained are comparable with those of other studies. The AEP also has the advantage of being diagnosis-independent and having specific appropriate criteria, meaning that patients who do not meet them will most likely have been admitted inappropriately. The AEP is also the most widely used tool to evaluate the effectiveness of interventions aimed at reducing the inappropriateness of hospital utilization.^30–33^

The prevalence of inappropriate admissions of 11.9% is consistent with the results obtained by Kouhestan et al. in a meta-analysis conducted in 2020, which found an incidence of 11.0%.^34^ The prevalence in this study also occupies an intermediate position compared with earlier studies, which registered variable frequencies depending on the care unit studied and the type of admission analyzed. Thus, incidence rates of inappropriate admissions range from 4.5% in Ochoa-Gomez et al., Spain, 2002, among patients in an emergency unit^15^ to 26.1% in Hammond et al., UK, 2009, in a unit for patients with long-term neurological impairment.^35^ In general, most related studies did not analyze the prevalence of admission inappropriateness for all units of a center simultaneously but tended to focus on the incidence in a single care unit.

Regarding patient characteristics, our study found no association between age and inappropriateness in our study. This differs from the results of the studies by Cordero et al. (in Portugal, 2001)^14^ and Coast et al. (in the UK, 1996)^36^, which observed a higher risk of inappropriate admission among older patients. Those studies did not carry out a bivariate analysis adjusted by type of admission, risk factors, comorbidities, and hospitalization as ours did, being that the most likeable reason that could explain our results.

Regarding the association between inappropriateness and the administrative nature of the admission, there was a higher prevalence among scheduled admissions (18.4% versus 8.7% among urgent admissions). However, the association disappeared when the number of intrinsic risk factors, clinical area of admission, age, and prognosis of the main illness were adjusted for. This association of inappropriateness with scheduled admissions was also found in the article by Campos et al. in 2006 in a pulmonology service in Spain.^12^ Along these lines, other inappropriateness studies focusing on patients from emergency departments, such as Smith et al. in the UK in 1997^37^ and Ochoa-Gomez et al.^15^, found slightly lower frequencies of 6.2% and 4.5%, respectively.

On the other hand, Capalbo et al. (in Italy, 2004) observed that inappropriate admissions were more frequent in 2-day hospitalizations than in 3-day ones^13^, concluding that inappropriate admission is related to shorter hospital stays. Our study found no significant differences in this respect (median 12 days versus 11; *p*=0.838). The explanation could be that we included all the hospitalized patients in a cross-sectional design without restriction by the length of stay. Capalbo et al.’s findings describe that in shorter hospital stays, there is usually a higher rate of inappropriateness, but, at the end, when the whole sample is included, other factors could extend these episodes, such as adverse events. The hospital where this study was conducted is a national reference for some surgeries and procedures, like neurosurgery ones or bone marrow transplants, that acted as outliers increasing the total length of stay. In recent research, the tendency has been to keep these cases in the analysis, and as this study did not intend to provide a specific analysis of the length of stay, we kept them in the sample.^38,39^

The causes of inappropriateness also varied depending on the setting studied. Rodríguez-Vera et al. in Spain in 2000 found that, in internal medicine units, premature admission was the main cause of inappropriateness^40^, while among patients referred from emergency units, the most frequent cause was outpatient management.^37^ In our study, premature admission was found in 78.4% of inappropriate scheduled admissions, and the possibility of outpatient management was found in 58.8% of urgent admissions, which is congruent with existing evidence.

When analyzing all IRFs in a multivariate model, we observed that the association between inappropriateness and intrinsic risk factors occurred more frequently among patients with one IRF, followed by those with two IRFs and a decrease in the strength of the association for those with more than three IRFs. This suggests an association between IRFs and inappropriate admission; however, it is not linear and is greater among patients with one or two comorbidities. These results are, however, difficult to compare since they have not been analyzed in previous studies.

Regarding the economic cost derived from inappropriate admissions calculated as 7.9 days of hospitalization, the total additional cost was €139,076.4, and the daily cost was €17,604.6. If these data were extrapolated to a cross-section of all patients admitted to the 70 hospitals with more than 500 beds in Spain, this level of inappropriate admission would cause a daily cost overrun of €1.2 million and an annual economic loss of €449.8 million. The clinical admission area had no impact on the total financial cost. However, admission in medical services had a higher impact due to avoidable hospitalization days (mean of 14.2 days vs 5.0 in surgical services). Inappropriate urgent admissions had double the financial cost of scheduled admissions (€96,805.3 versus €42,271.1), suggesting that improving the outpatient network would have a positive impact on the health system. However, these results have measurement and comparability limitations since economic estimates have traditionally been made on the basis of the individual study of the appropriateness of each day of hospital stay, and our estimation does not consider the specific procedures and treatments that the patient received during his hospitalization.^33,41^ Also, some cost could be overestimated, such as those related to patients in the hospital waiting surgical procedures, who may not consume many resources.

Another limitation would be related to the characteristics of the AEP itself, which was designed to minimize value judgments and increase concordance between reviewers. For example, the following items would meet the criteria for an appropriate admission: (1) the administration of intravenous medication; (2) the indication of any scheduled/urgent procedure within the next 24 h that required general or regional anesthesia, or equipment or materials available only for inpatients. However, the underlying necessity of the route of administration or the procedure was not assessed. This phenomenon could underestimate inappropriateness.

Another one would be that the information for the AEP was obtained by only one reviewer. Nevertheless, AEP is the most used validated tool in the literature and provides better data quality than a panel of experts. Another limitation is related to the collection of social determinants of health. Previous evidence focused on this relationship and we pretended to provide a new approach to exploring whether there was any association between the patient’s comorbidities and inappropriateness. Future research should combine the study of both fields in order to improve the evidence of the inappropriateness of admissions. Finally, although the analyzed center is one of the main ones in the region, another limitation would be that this study was a single center and some local practice patterns could affect the results. However, some important findings in this research allow the development of future multi-center analysis that could provide a more accurate approach to some of our results.

Among the strength of this study, the following are especially relevant: (1) this is the first study that uses the AEP in an epidemiological perspective, analyzing how IRFs influence hospital admissions; (2) to the best of our knowledge, this study is also a pioneer in applying a cross-sectional design to all patients admitted to a hospital and in providing a prevalence value for inappropriate admission; (3) last, the patients’ IRFs included were collected using the same criteria established by studies of recognized prestige in the field of patient safety, such as ESHMAD^17,18,42^ (based on the methodology of *The Harvard Medical Practice Study*)^19^ and EPINE.^20^

Finally, the inappropriateness of admissions results in a problem of great magnitude within health care due to its frequency and economic impact. In addition, the results place patients with an intermediate number of comorbidities as those who are most likely to suffer from this phenomenon and are a good target to mitigate its impact. Future research, with a longitudinal design, should delve into the consequences of inappropriate admissions in the form of harm to the patient and the factors that cause a patient with intermediate comorbidities to be associated with inappropriateness. This new focus for understanding the causes underlying this type of inappropriateness would be able to drive strategies for improving and optimizing resources.^6,41^

## CONCLUSIONS

The prevalence of inappropriate admissions is similar to the incidence found in previous studies conducted in several countries. Patients with ≥1 IRF had a higher prevalence of inappropriate admission, showing a nonlinear association.

Unlike other studies, age, sex, and the administrative status of admission showed no association when adjusted for the number of comorbidities and prognosis of the disease-causing admission. Among scheduled admissions, the prevalence of inappropriate admissions was higher and was the most frequent cause of premature admission, whereas, among urgent admissions, the most frequent cause was the possibility of managing the patient in the outpatient setting.

Inappropriate admissions have a great financial impact due to the associated days of hospitalization, mainly from urgent inappropriate admissions, which suggests that improving the outpatient care network could mitigate this.

## Supplementary Information

ESM 1 (DOCX 20 kb)

## References

[1] **Hopkins A, Fitzpatrick R, Foster A, et al.** What do we mean by appropriate health care? Report of a working group prepared for the Director of Research and Development of the NHS Management Executive. Quality and Safety in Health Care. 1993;2:117–23. 10.1136/qshc.2.2.117 PMC1055096 10131631

[2] **Chassin MR, Galvin RW**. The urgent need to improve health care quality. Institute of Medicine National Roundtable on Health Care Quality. JAMA. 1998;280:1000–5. 10.1001/jama.280.11.1000 9749483

[3] **Lavis JN, Anderson GM**. Appropriateness in health care delivery: definitions, measurement and policy implications. CMAJ. 1996;154:321–8. PMC1487507 8564901

[4] **Peiró S, Meneu R**. Revisión de la utilización. Definición, Concepto y Métodos. Rev Calidad Asistencial 1997;12: 122-136.

[5] **Shrank WH, Rogstad TL, Parekh N.** Waste in the US Health care system: estimated costs and potential for savings. JAMA. 2019;322:1501–9. 10.1001/jama.2019.13978 31589283

[6] **Arab-Zozani M, Pezeshki MZ, Khodayari-Zarnaq R, Janati A**. Inappropriate rate of admission and hospitalization in the Iranian hospitals: a systematic review and meta-analysis. Value Health Reg Issues. 2020;21:105–12. 10.1016/j.vhri.2019.07.011 31704488

[7] **Liu W, Yuan S, Wei F, Yang J, Ma J**. Inappropriate admissions of the cardiology and orthopedics departments of a tertiary hospital in Shanghai, China. PLoS ONE. 2018;13:e0208146. 10.1371/journal.pone.0208146 PMC6300262 30566422

[8] **Lang T, Davido A, Logerot H, Meyer L**. Appropriateness of admissions: the French experience. Int J Qual Health Care. 1995;7:233–8. 10.1093/intqhc/7.3.233 8595460

[9] **Fellin G, Apolone G, Tampieri A, Bevilacqua L, Meregalli G, Minella C, et al.** Appropriateness of hospital use: an overview of Italian studies. Int J Qual Health Care. 1995;7:219–25. 10.1093/intqhc/7.3.219 8595458

[10] **Restuccia JD**. The evolution of hospital utilization review methods in the United States. Int J Qual Health Care. 1995;7:253–60. 10.1093/intqhc/7.3.253 8595463

[11] **Gertman PM, Restuccia JD**. The appropriateness evaluation protocol: a technique for assessing unnecessary days of hospital care. Med Care. 1981;19:855–71. 7196975

[12] **Campos Rodríguez F, De la Cruz Morón I, López Rodríguez L, Díaz Martínez A, Tejedor Fernández M, Muñoz Lucena F**. Appropriateness of hospital admissions to a pulmonology department. Arch Bronconeumol. 2006;42:440–5. 10.1016/s1579-2129(06)60566-1 17040659

[13] **Capalbo G, D’Andrea G, Volpe M, Cambieri A, Cicchetti A, Ricciardi G**. Appropriateness evaluation of short hospital admissions using Appropriateness Evaluation Protocol (Italian version): experience of a teaching hospital. Ann Ig. 2004;16:759–65. 15697006

[14] **Cordero A, Aguila J, Massalana A, Escoto V, Lopes L, Susano R**. Appropriateness admissions to the Department of Internal Medicine of the Hospital de Santa Luzia (Elvas) evaluated by the AEP (Appropriateness Evaluation Protocol). Acta Med Port. 2004;17:113–8. 15921640

[15] **Ochoa-Gómez J, Villar Arias A, Ramalle-Gómara E, Carpintero Escudero JM, Bragado Blas L, Ruiz Azpiazu JI.** Adecuación de los ingresos hospitalarios urgentes. Anales de Medicina Interna. 2002;19:8–10. 12420627

[16] **Liu W, Yuan S, Wei F, Yang J, Zhu C, Yu Y, et al.** Inappropriate hospital days of a tertiary hospital in Shanghai, China. Int J Qual Health Care. 2017;29:699–704. 10.1093/intqhc/mzx091 28992148

[17] **Valencia-Martín JL, Vicente-Guijarro J, San Jose-Saras D, Moreno-Nunez P, Pardo-Hernández A, Aranaz-Andrés JM, et al.** Prevalence, characteristics, and impact of Adverse Events in 34 Madrid hospitals. The ESHMAD study. Eur J Clin Invest. 2022;e13851. 10.1111/eci.13851 PMC9787492 35909351

[18] **San Jose-Saras D, Valencia-Martín JL, Vicente-Guijarro J, Moreno-Nunez P, Pardo-Hernández A, Aranaz-Andres JM.** Adverse events: an expensive and avoidable hospital problem. Ann Med. 2022;54:3157–68. 10.1080/07853890.2022.2140450 PMC9665082 36369717

[19] **Brennan TA, Leape LL, Laird NM, Hebert L, Localio AR, Lawthers AG, et al.** Incidence of adverse events and negligence in hospitalized patients. Results of the Harvard Medical Practice Study I. N Engl J Med. 1991;324:370–6. 10.1056/NEJM199102073240604 1987460

[20] Estudio de Prevalencia de Infecciones Nosocomiales. Informe España. 2019. Sociedad Española de Medicina Preventiva, Salud Pública e Higiene. [cited 2022 February 25]. Available from: https://epine.es/api/documento-publico/2019%20EPINE%20Informe%20Espa%C3%B1a%2027112019.pdf/reports-esp. Accessed 25 February 2022.

[21] European Centre for Disease Prevention and Control. Point prevalence survey of healthcare-associated infections and antimicrobial use in European acute care hospitals :2011 2012. [Internet]. LU: Publications Office; 2013 [cited 2022 Apr 22]. Available from: https://data.europa.eu/doi/10.2900/86011 Accessed 22 April 2022.

[22] StataCorp. 2019. Stata Statistical Software: Release 16. College Station, TX: StataCorp LLC.

[23] **Gupta S, Taylor N, Selvakumar D, Harnett PR, Wilcken N, Lee CI.** Retrospective imaging audit and cost analysis of medical oncology inpatients admitted to Westmead Hospital. Intern Med J. 2014;44:1235–9. 10.1111/imj.12565 25169081

[24] **Siu AL, Sonnenberg FA, Manning WG, Goldberg GA, Bloomfield ES, Newhouse JP, et al.** Inappropriate use of hospitals in a randomized trial of health insurance plans. N Engl J Med. 1986;315:1259–66. 10.1056/NEJM198611133152005 3773939

[25] **Guilé R, Leux C, Paillé C, Lombrail P, Moret L**. Validation of a tool assessing appropriateness of hospital days in rehabilitation centres. Int J Qual Health Care. 2009;21:198–205. 10.1093/intqhc/mzp008 19251730

[26] **Esmaili A, Ravaghi H, Seyedin H, Delgoshaei B, Salehi M**. Developing of the Appropriateness Evaluation Protocol for Public Hospitals in Iran. Iran Red Crescent Med J. 2015;17:e19030. 10.5812/ircmj.19030 PMC4441772 26019898

[27] **Leung LP, Fan KL**. Who should be admitted to hospital? Evaluation of a screening tool. Hong Kong Med J. 2008;14:273–7. 18685159

[28] **Peiro S, Meneu R, Portella E et al.** Validez del protocolo de evaluación del uso inapropiado de la hospitalización. Medicina Clínica.1996 [Internet]. [cited 2022 Feb 11]. Available from: https://www.uv.es/~docmed/documed/documed/668.html. Accessed 11 February 2022. 8754481

[29] **Lee C, Kim SJH, Lee C, Shin E**. Reliability and validity of the appropriateness evaluation protocol for public hospitals in Korea. J Prev Med Public Health. 2019;52:316–22. 10.3961/jpmph.19.125 PMC6780293 31588701

[30] **Kossovsky MP, Chopard P, Bolla F, Sarasin FP, Louis-Simonet M, Allaz AF, et al.** Evaluation of quality improvement interventions to reduce inappropriate hospital use. Int J Qual Health Care. 2002;14:227–32. 10.1093/oxfordjournals.intqhc.a002614 12108533

[31] **Moya-Ruiz C, Peiró S, Meneu R**. Effectiveness of feedback to physicians in reducing inappropriate use of hospitalization: a study in a Spanish hospital. Int J Qual Health Care. 2002;14:305–12. 10.1093/intqhc/14.4.305 12201189

[32] **Antón P, Peiró S, Aranaz JM, Calpena R, Compañ A, Leutscher E, et al.** Effectiveness of a physician-oriented feedback intervention on inappropriate hospital stays. J Epidemiol Community Health. 2007;61:128–34. 10.1136/jech.2005.040428 PMC2465655 17234871

[33] **Soria-Aledo V, Carrillo-Alcaraz A, Flores-Pastor B, Moreno-Egea A, Carrasco-Prats M, Aguayo-Albasini JL**. Reduction in inappropriate hospital use based on analysis of the causes. BMC Health Serv Res. 2012;12:361. 10.1186/1472-6963-12-361 PMC3507908 23075150

[34] **Kouhestan SM, Hashempour R, Raei B, Chivaee D, Safari H**. Prevalence and determinants of inappropriate admission and hospitalization in Iran: A systematic review and meta-analysis. Med J Islam Repub Iran. 2020;34:2. 10.34171/mjiri.34.2 PMC7139265 32284926

[35] **Hammond CL, Phillips MF, Pinnington LL, Pearson BJ, Fakis A**. Appropriateness of acute admissions and last in-patient day for patients with long term neurological conditions. BMC Health Serv Res. 2009;9:40. 10.1186/1472-6963-9-40 PMC2653500 19250523

[36] **Coast J, Peters TJ, Inglis A**. Factors associated with inappropriate emergency hospital admission in the UK. Int J Qual Health Care. 1996;8:31–9. 10.1093/intqhc/8.1.31 8680815

[37] **Smith HE, Pryce A, Carlisle L, Jones JM, Scarpello J, Pantin C**. Appropriateness of acute medical admissions and length of stay. J R Coll Physicians Lond. 1997;31:527–32. PMC5420957 9429190

[38] **Hughes AH, Horrocks D, Leung C, Richardson MB, Sheehy AM, Locke CFS**. The increasing impact of length of stay ‘outliers’ on length of stay at an urban academic hospital. BMC Health Serv Res. 2021;21:940. 10.1186/s12913-021-06972-6 PMC8427900 34503494

[39] **Kerguelen C, de la Hoz-Valle JA**. The impact of outliers on available resources in a teaching hospital in Colombia. Am J Manag Care. 2021;27:e365. 10.37765/ajmc.2021.88778 34784143

[40] **Rodríguez-Vera FJ, Alcoucer Díaz MR, Rodríguez Gómez FJ, Martínez García T, Colchero Fernández J, Pujol de la Llave E.** Inappropriate admissions to the Department of Internal Medicine evaluated by the AEP (Appropriateness Evaluation Protocol). An Med Interna. 2000;17:47–8. 10730407

[41] **Soltani S, Hoseini Kasnavieh M, Shaker H, Abbasian A, Amanollahi A, Tahmasebi A.** Evaluation of Inappropriate Admission and Hospitalization According to Appropriateness Evaluation Protocol and Estimation of Direct Financial Burden. Shiraz E-Med J [Internet]. 2019 [cited 2022 Jan 10];20. Available from: https://brief.land/semj/articles/87870.html#abstract. Accessed 10 January 2022.

[42] **Aranaz-Andrés JM, Limón R, Mira JJ, Aibar C, Gea MT, Agra Y, et al.** What makes hospitalized patients more vulnerable and increases their risk of experiencing an adverse event? Int J Qual Health Care. 2011;23:705–12. 10.1093/intqhc/mzr059 21896634

