# Supplementary material for: Inappropriate Hospital Admission According to Patient Intrinsic Risk Factors: an Epidemiological Approach
Source: J Gen Intern Med. 2023 Jan 30;38(7):1655–63. doi: 10.1007/s11606-022-07998-0 (PMC10212828; doi:10.1007/s11606-022-07998-0)
Supplement: Supplementary file 1 — (DOCX 20 kb) [file 11606_2022_7998_MOESM1_ESM.docx]

**Figure S1: Appropriateness Evaluation Protocol**

| **INAPROPRIATENESS OF ADMISSION IN ADULT PATIENTS** | |
| --- | --- |
| **Patient’s clinical situation** | |
|  | 1. Sudden loss of consciousness or disorientation (coma or numbness) |
|  | 2. Pulse < 50 beats or >140 beats per minute |
|  | 3. Blood pressure: Systolic <90 or >200 mmHg; Diastolic <60 or >120 mmHg |
|  | 4. Sudden loss of vision or hearing |
|  | 5. Sudden paralysis of any part of the body |
|  | 6. Persistent fever: 38ºC taken in the mouth (>38.5ºC elsewhere) for more than 5 days. |
|  | 7. Active bleeding |
|  | 8. Severe electrolyte or blood gas disturbances: Na < 123 mEq/l or > 156 mEq/l; K < 2.5 mEq/l or > 6 mEq/l; CO2 combining power (except chronic anomalies < 20 mEq/l CO2 or > 36 mEq/l CO2; arterial pH < 7.30 or > 7.45 |
|  | 9. Electrocardiographic evidence of acute ischemia. |
|  | 10. Suture dehiscence or evisceration. |
| **Clinical services** | |
|  | 11. Administration of intravenous medication and/or fluid replacement (does not include nasogastric tube for feeding) |
|  | 12. Scheduled/urgent surgery or procedure within the next 24 hours that requires: a) general or regional anesthesia, or b) equipment or materials available only for inpatients |
|  | 13. Vital signs monitoring every 2 hours or more often (including telemetry or cardiac monitoring) |
|  | 14. Chemotherapy that requires continuous observation for the treatment of life-threatening toxic reactions |
|  | 15. Administration of intramuscular antibiotics at least every 8 hours. |
|  | 16. Use of intermittent or continuous respirator, at least every 8 hours |
| **If no box is filled in, admission is inappropriate** | |
|  |  |
|  |  |
|  |  |
| **Causes of inappropriateness** | |
|  | 1. The case could be dealt with as an outpatient. |
|  | 2. Same as 1 but the patient lives too far from the hospital for outpatient care |
|  | 3. Same as 1 but it was not possible to schedule the patient, or it was too late. |
|  | 4.0 The patient needs lower-level institutional care |
|  | 4.1 Needs care typical of a hospital for chronic illnesses |
|  | 4.2 The patient needs care typical of an assisted living facility |
|  | 4.3 The patient needs care in a minimum care nursing home |
|  | 5. Premature admission |
|  | 6. Others |

**Table 1: Distribution and Inappropriateness for each IRF.**

| **Table S1. Distribution and Inappropriateness for each IRF** | | | | |
| --- | --- | --- | --- | --- |
|  | **Total** | **Inappropriateness** | |  |
|  | **n (% In total)** | **n** | **% (CI 95%)** | **p-value** |
| **Intrinsic Risk Factors** | | | | |
| Cardiovascular disease | 339 (57.2) | 36 | 10.6 (7.5 to 14.4) | 0.241 |
| Mobility issues | 227 (38.3) | 32 | 14.1 (9.8 to 19.3) | 0.210 |
| Neoplasia | 202 (34.1) | 18 | 8.9 (5.4 to 13.7) | 0.099 |
| Hypoalbuminaemia | 195 (33.1) | 15 | 7.6 (4.4 to 12.4) | 0.025* |
| Diabetes | 170 (28.7) | 21 | 12.4 (7.8 to 18.3) | 0.880 |
| Obesity | 137 (23.0) | 19 | 13.9 (8.6 to 20.8) | 0.436 |
| Sensory deficit | 136 (22.9) | 17 | 12.3 (7.5 to 19.3) | 0.884 |
| Coma | 135 (22.8) | 13 | 9.6 (5.2 to 15.9) | 0.340 |
| Chronic pulmonary disease | 97 (16.4) | 12 | 12.4 (6.6 to 20.6) | 0.895 |
| Active smoker | 96 (16.1) | 6 | 6.3 (2.3 to 13.1) | 0.060 |
| Inmunodeficiency | 43 (7.2) | 3 | 6.9 (1.5 to 19.1) | 0.462 |
| Pressure ulcers | 33 (5.6) | 3 | 9.1 (1.9 to 24.3) | 0.786 |
| Cirrhosis | 24 (4.0) | 5 | 20.8 (7.1 to 42.2) | 0.192 |
| Neutropenia | 20 (3.4) | 1 | 5.0 (0.1 to 24.9) | 0.494 |
| Kidney failure | 14 (2.7) | 0 | NA | 0.394 |
| P for difference of percentages, Chi2 test (if parametric test conditions) and Fisher exact test (non-parametric). | | | | |
| *p<0,05; **p<0,001 | | | | |
